# Genome-Wide Analysis of Intergenic Regions of *Mycobacterium tuberculosis* H37Rv Using Affymetrix GeneChips

**DOI:** 10.1155/2007/23054

**Published:** 2007-09-13

**Authors:** Li M Fu, Thomas M Shinnick

**Affiliations:** 1 Pacific Tuberculosis and Cancer Research Organization, 8 Corporate Park, Suite 300, Irvine, CA 92606, USA; 2 Centers for Disease Control and Prevention, Atlanta, GA 30333, USA

## Abstract

Sequencing the complete genome of *Mycobacterium tuberculosis* H37Rv is a major milestone in the genome project and it sheds new light in our fight with tuberculosis. The genome contains around 4000 genes (protein-coding sequences) in the original genome annotation. A subsequent reannotation of the genome has added 80 more genes. However, we have found that the intergenic regions can exhibit expression signals, as evidenced by microarray hybridization. It is then reasonable to suspect that there are unidentified genes in these regions. We conducted a genome-wide analysis using the Affymetrix GeneChip to explore genes contained in the intergenic sequences of the *M. tuberculosis* H37Rv genome. A working criterion for potential protein-coding genes was based on bioinformatics, consisting of the gene structure, protein coding potential, and presence of ortholog evidence. The bioinformatics criteria in conjunction with transcriptional evidence revealed potential genes with a specific function, such as a DNA-binding protein in the CopG family and a nickle binding GTPase, as well as hypothetical proteins that had not been reported in the H37Rv genome. This study further demonstrated that microarray-based transcriptional evidence would facilitate genome-wide gene finding, and is also the first report concerning intergenic expression in *M. tuberculosis* genome.

## 

[1,2,3,4,5,6,7,8,9,10,11,12,13,14,15,16,17,18,19,20]
